# Bibliographical Notices

**Published:** 1836-10-01

**Authors:** 


### Bibliographical Notices.

On Deformities of the Chest. By Mr. Coulson.

This little brochure is not on the hacknied subject of spinal distortion, but on "lateral and anterior compression" of the thorax. In the former, the sternum is prominent, and the sides of the chest are flattened in. The individual is said to be chicken-breasted. It is now more than ten years since Dupuytren published a paper in the "*Repertoire d'Anatomie*" on this distortion which, though sometimes congenital, is generally traceable to weakness of constitution, scrofula, low, damp and unhealthy habitations, bad cloathing and unwholesome food. This state must, of course, be unfavourable to the functions of circulation, respiration, &c. and it would be very desirable to counteract the deformity if possible. Dupuytren draws a distressing picture of the consequences of laterally flattened chest in hooping-cough—especially when enlarged tonsils accompany the deformity, as it usually does.

The other species of distortion is a flattening in of the sternum, or anterior depression. The external appearances are here the reverse of what they are in the other species. The sternum is hollow, and the sides are bulged out.

"The constitutional remedies for both deformities are the same. We must order nutritious diet; regulate the digestive function; and perhaps employ, as Dr. Copland recommends, the artificial salt-water bath, with a very large proportion of salt, at a temperature suited to the peculiarities of the case (or preferably sea-bathing),—in short, we must make use of all those means by which the system can be duly kept up.

I concur, however, with Dupuytren, in thinking that a strengthening regimen, and the use of tonics should be used with all the moderation required by the embarrassment of respiration and the disorder of the circulation, which might be increased, and even rendered dangerous, by a regimen and remedies of too tonic a nature, or given in too great a quantity.

Whatever mode of treatment be adopted, there can be no doubt that a pure air is requisite to its success. My trust, however, is in other than constitutional remedies."

The remedy of Dupuytren is as follows:—

"Of all those which I have used," says Dupuytren, "I have found none more efficacious than exercises adapted to strengthen the muscles which extend from the arms and shoulders to the chest, and especially than frequent pressure upon the sternum from before backwards."

Mr. Coulson has used this remedy in lateral depression, or chicken-breast, with success; but he prefers voluntary exercise to passive pressure.

"'The object and result of the exercises I recommend,' says Dupuytren, 'is to raise up the sides of the chest, to separate them, to make them turn outward, and finally to restore them to their natural conformation. There is no exercise better adapted for this purpose than that which obliges persons labouring under this malformation, to raise a weight suspended to a cord passed through two pulleys, by the aid of their arms and hands, during several hours daily. The end of the cord to be grasped should be fastened to the middle of a lever to be taken hold of by the two hands, the other extremity supporting a weight proportioned to the strength of the individual. The individual standing upright, or even rising on tiptoe, to reach the lever placed at the extremity of the cord, seizes it with both his hands; and employing the power of the muscles of the fore-arm, arm, neck and chest, to bend the head, chest and body downwards at the same time, must raise the weight at the other extremity of the cord, and alternately employ the flexor muscles to raise the weight, and the extensors to straighten the body. If it be true—and there is no doubt of it—that there exist between the bones and muscles relations of conformation and action, so that the latter always tend to act upon the former, in such a manner as to bring them to their first and fixed shape; it is certain that the exercise we have just described, will, by directing the efforts of the muscles upon the bones of the chest, gradually bring the sides of this cavity to an improved form.'

Perhaps the best mode of overcoming the depression by developing muscular action and power, similarly observes Dr. Copland, ' is to cause the child to raise weights, by means of ropes and pullies placed a considerable height over his head; so that, by taking hold of the rope with both hands raised above the head, and pulling it downwards, the muscles may be brought into action, and the parietes of the chest thereby dilated.'

Participating in these views, I observed in my former paper, that ' the child should stand erect, and *carry the arms as far backwards as it can;*' and, to the latter injunction, I still attach much importance. With a vague impression, however, that the exercise prescribed by Dupuytren was not the best devised, since in it the weight of the body and the contraction of the abdominal muscles must depress the ribs, I observed, that ' the use of the dumb-bells is a good exercise,' and added, that ' any exercise, indeed, is good, by which the scapulæ are approximated towards each other, and the arms carried backwards.'

I am now satisfied that carrying the arms backward and approximating the scapulæ, without at the same time depressing the ribs by the weight of the body and the action of the abdominal muscles, is all that is requisite. I object, however, to the employment of dumb-bells for that purpose, on account of the jerk which they produce, the involuntary action (that is, beyond a certain extent) which that implies, and even the danger which it produces; and I deem the Indian Exercises, first described in this country by Donald Walker, in his ' Exercises for Ladies,' as greatly preferable to all others, both in these and in every other deformity of the chest. They raise up the ribs and sternum, without the slightest counteracting tendency to depress them, and they give the fullest expansion to the chest.

By these various means, if properly persevered in, the chest returns to its natural shape, and the whole system becomes invigorated."

Some appropriate cases and plates illustrate this little volume, which we recommend to our surgical brethren. Mr. Coulson seems to have devoted much attention to the subject, and has handled it well.

---

AN ENQUIRY INTO THE PATHOLOGY, CAUSES, AND TREATMENT OF PUERPERAL FEVER. By G. MOORE, Esq.

This Essay gained the Fothergillian Medal, annually offered by the London Medical Society; and it is a curious circumstance that it is, almost wholely and solely, an Analytical Review of the principal authors who have written on the subject of puerperal fever in this and other countries. The essay is truly eclectic, and would have made a capital article in the Cyclopædia of Practical Medicine. There is, as the author candidly acknowledges, very little originality in the work, " except that of arrangement and combination." The work is, however, much more valuable, and will prove much more useful, than any original essay could have proved. The compilation is very judicious, and the labour expended in searching out materials from musty, often-forgotten tomes, and sifting the grain from the chaff, must have been very considerable. An Analytical Review itself, the volume is totally insusceptible of analysis. We think it ought to be in the hands of most practitioners, as an excellent resumé of all that has been said and done respecting a most formidable disease. We shall select a specimen of the composition of this volume—nearly at random.

" Morbid anatomy discloses but little concerning puerperal fever, by which the physician can be guided in the employment of therapeutic measures. The varieties which it presents, as far as regards all practical purposes, are much better ascertained by symptomatology, than by dissection. In fact it would be almost impossible to determine what particular affection of tissue is associated with particular symptoms, since every variety of structural alteration may exist together in the same case. Yet, necrotomy is of great value, as auxiliary to the consideration of this disease, as it sufficiently accounts for the failure of remedy, and proves that unless the destructive activity of morbid processes be speedily arrested, the lesions of structure produced are often such, as neither the restorative powers of nature can repair, nor those of medicine reach.

The disease has been divided into varieties almost as numerous as its successive symptoms would allow, but we have good authority to conclude that all the varieties may be safely reduced to three—these are, inflammatory, synochoid, and adynamic.

If all the circumstances already enumerated, be viewed in their proper relation to each other, it will appear, that whether the disease shall be deemed inflammatory, synochoid, or adynamic, will depend not so much on the nature of the structure most implicated, as on the constitutional state of the patient at the period of attack. The synochoid and adynamic varieties, although of course more speedily and more extensively evincing alteration in the condition of the blood, and also in the fluids secreted from it, than in the stage of simple inflammation, are nevertheless as intimately associated with structural disorder, as that which is called inflammatory. Metritis, peritonitis, and uterine phlebitis, are certainly as often connected with one form of fever as the other, but undoubtedly the rapidity with which sanguineous deterioration and consequent adynamia take place, will greatly depend on the nature, extent, and importance of the structure primarily involved. Hence it would appear that it may best be treated, at its commencement at least, as simple synochial fever, with the understanding that if it be not arrested in its first stage, the condition of the body is such, that some organic change must inevitably follow, which no art can control. Even if we were obliged to conclude, which we are not, that it invariably has its source in specific contagion, yet the malady must be encountered according to the mode of its manifestation, and not simply according to the notion we may entertain concerning its exciting cause, although of course that notion will somewhat influence our procedure, but merely in as far as to remove a consequence first requires the removal of the cause. The disease, however, is the same, by whatever cause produced, and the withdrawal of the cause is not sufficient to effect a cure, for the continued action of the originating impulse, is not by any means necessary to effect the establishment and development of disorder, since the system having acquired a morbid momentum is disposed to proceed morbidly, according to its own laws, and not according to any specific influence derived from the determining impulse. Our remedies must act on the medium through which the morbid influence itself acts, that is, the nervous system considered as a whole.

The practical varieties are therefore to be viewed as merely presenting different manifestations of the patient's condition of system at the period of attack. They are marked by certain symptoms which, from their prominence, claim the chief attention, and must, of course, be met according to their several indications; but, both symptoms and morbid appearances, evince the direct tendency of the disorder, to the rapid establishment of violent and extensive inflammation, particularly in the uterus, its veins and appendages, and in the peritoneum, or in any part which may then be subjected to violence.

Here the advantage of correct theory, concerning inflammation, would be experienced. But as the theory of its cause involves a problem not yet resolved, we must be guided by general experience in its treatment. The principal object is to resist the incursion of this inflammation by the most vigorous measures at its very commencement. This is best accomplished by abstraction of blood, either by venesection, by cupping, or by leeches. Venesection is most frequently adopted."

ON THE ANTIDOTAL TREATMENT OF THE EPIDEMIC CHOLERA. By JOHN PARKIN, late of the Honourable East India Company's Service, &c.

The cholera storm has blown over here, and people are putting their heads out of doors, and wondering that so small a matter could have made so great a noise. The Cholera Boards have dissolved like

"Snaw i' the hot sun, my dear,"

and the worthy gentlemen who recommended us to put a cross upon our doors, drink our soup through the window in buckets, and place our parishes under the college-militant, look foolish and uneasy when the subject is mentioned, and would willingly forget that they took such especial pains to be ridiculous. The Lancet and Gazette congratulate themselves no more on belonging to the same party, the former no longer promising a lordship to sucking contagionists, and the latter ceasing to call down Heaven's vengeance and the mob's on all who opposed quarantine restrictions.* Peace to the manes of the ultra-contagionists, for they are fairly under ground.

Who does not recollect the cloud of pamphlets that obscured the sky, when the cholera was in existence. Reports, propositions, cures, crowded our table till it groaned. Who does not remember too the sudden disappearance of octavo, duodecimo, and octo-decimo, with the occasion. So soon as the disease died, the mortality amongst the pamphlets was quite frightful. The authors sank into a sudden collapse, and when the publisher's bills were at last presented, the blueness of the features was most striking.

Once or twice since that period our slumbers have been broken, by a new cholera pamphlet. Like a shadow it has come, and so departed, none seeming to heed either book or book-maker. Mr. Parkin has just sent forth a slim octavo, the sight of which occasioned these recollections.

Now don't, reader, be alarmed. We have no more intention of reviewing this same octavo, than of giving an account of Halley's comet. Pray then be easy and hear what we have to say.

Mr. Parkin is satisfied that carbon or carbonic acid is an antidote for the poison of cholera. He has told the world so several times already, but, as it wouldn't believe him, he has written a book. If that fails to convince, he must adopt the expedient of my Uncle Toby, and tell this generation to "go and be ———."

He says it is unimportant what his theory may be; and we agree with him. Like Mr. Parkin, we are practical folks, and if the thing is a specific, we care not under what hypothesis it was brought forth. Carbonic acid *is* an antidote. Mr. Parkin, who has tried it more than any body else, and, of course, knows more about it, has no hesitation in saying so. That is enough.

"When carbonic acid has been given in any case in which the stomach is alone affected, the effect of the medicine, according to my experience, has been to relieve the symptoms almost immediately. The nausea is speedily dissipated, the giddiness and faintness disappear, and the sensation of burning and heat at the pit of the stomach is no longer felt, or complained of, after two or three doses of the medicine.

But the most remarkable circumstance is that which has attended the employment of the same agent during the stage of diarrhœa; of that relaxation of the bowels which is, almost always, the precursor of the severe form of cholera. Of the numerous cases in which I have given the carbonic acid, at this period, it has invariably arrested, with some few exceptions to be explained hereafter, the morbid process; at a longer period, it is true, but still at a regular and certain interval.

Neither is the remedy less useful in

* These are facts. The Lancet gravely promised that Parliament would grant a *title* to the discoverer of a specific for cholera. The Gazette's thunder against the anti-alarmists was peculiarly rumbling, and would have turned the best beer in Dennis Brulgruddery's cellar.

what has been termed the evacuant, or second stage of the disease, characterized by rice-water evacuations. In these cases, the irritability of the stomach is speedily relieved, and the vomiting ceases soon after the first, or at most the second, dose of the medicine —while the relaxation of the bowels is also arrested, if not with the same, with almost as great celerity as in the former instance.

But it is in the commencement of collapse that the efficacy of the remedy is best observed, and its *modus operandi* the most apparent. Not only is the vomiting immediately arrested, as in the previous stage; but the thirst, heat, and burning sensation at the pit of the stomach, disappear almost as speedily. During a repetition of the medicine, the spasms which generally prevail at this period are effectually relieved; the evacuations from the bowels become less abundant and less frequent; the depression of the system is removed; and the other symptoms characteristic of this stage vanish by degrees—so as frequently to leave the patient comparatively free from all ailment after the administration of only five or six doses of the medicine."

If carbonic acid relieves diarrhœa, rice-water evacuations, and collapse, we do not see how it can be considered to be any thing but an antidote. It is the very remedy that is required. But how are we to give it.

"Thirty grains of the powdered carbonate or bicarbonate (not the subcarbonate) of soda, or potash, should be placed in a large tumbler, to which is to be added a dessert spoonful of any simple syrup, mixing the two ingredients together so as to form a homogeneous mass. Then take twenty grains of citric or tartaric acid, and dissolve it in a wine-glass full of water, when the solution is to be poured on the contents of the tumbler, and the mixture drank off immediately, before the effervescence has subsided. If more convenient, or when to be obtained, lemon-juice may be substituted for the citric or tartaric acid, in the proportion of two table spoonsful of lemon-juice to the same quantity of soda or potash. As the object, in giving the syrup, is to render the mixture more tenacious, and prevent the gas escaping so rapidly as would otherwise be the case, it is not necessary to use any, or at least, so great a quantity in the latter as in the former instance."

Thus effervescing draughts are a medium of conveying carbonic acid, and effervescing draughts, have we know been always given, more or less. To be sure other people have given them under precisely an opposite notion, but that is immaterial. They gave a good remedy with a wrong view; but still they gave it. Even the immortal Stevens gave saline draughts at Cold-bath Fields. How successful his practice was, poor Sir David Barry's report has told. It not only cured cholera, but prevented it, and in a few hours after the intelligent Doctor had reported the frightful state of the prison, Sir David found hardly an instance of cholera in it.

We do not think it necessary to give Mr. Parkin's directions for the administration of the carbonic acid in full. Those who feel anxiety upon the subject may refer to his book for information. He is fully convinced that carbonic acid *is* an antidote to cholera, and if the profession is incredulous, he may safely wash his hands of the matter, and reflect with satisfaction that he has done his best to persuade it.

---

The Practical Anatomy and Elementary Physiology of the Nervous System; designed for the Use of Students in the Dissecting-Room. By F. Le Gros Clark, Demonstrator of Anatomy in St. Thomas's Hospital. 12mo, pp. 367. Longman's London, 1836.

The alteration which has taken place in the turn and temper of men's minds is so obvious, that we need not dwell on it. The taste for abstract speculation and for theoretical reasoning has sensibly declined, and facts and strict inductions from them are universally demanded.

It is not surprising if anatomy has felt the influence of so great and so important a change—on the contrary, it is strange that it did not receive a more decided impression long ago. The fact is, that rigorous observation and description prevailed on the Continent, and especially in France, before they were extensively practised here. The reason must be sought in the difficulties that, till lately, obstructed dissection in this country, and the consequent disposition to generalize, that was occasioned by the want of opportunities for frequent and minute investigations.

It was natural, under such circumstances, to try to make the most of what could be readily obtained, and to hug the belief, that the broad facts of anatomy were all that were important. The doctrine was popular and the practice general, and the convenient excuse which they offered, both to the teacher and the pupil, contributed, we may be sure, to promote their diffusion. The former said that minute anatomy was useless—the latter found it was a bore—the indolence of both promoted a notion, which first originated in the pocket—and gradually it came to be a matter of faith, which was not so much as challenged.

Manuals of all sorts were of course in vogue, and every fresh author proposed his vade-mecum, as a still straighter and shorter cut to knowledge than its predecessors. These manuals tended to discourage accurate investigations. How could they do else? Their very existence depended on the taste for superficial study. The author always affected to direct attention to *practical* points—to things which would be of use. "Do not (said he to the willing student) trouble yourself with minutiæ, which can never be of service to you, learn the main facts, your knowledge of which can be applied to the cases that will daily come before you." The student listened, arrectis auribus, to the flattering tale, and, as he was told to attend to the main facts, he took good care that he would go no farther (he seldom went so far) than his preceptor wished. That was the golden age of hand-books; but an iron one was coming.

While teacher and taught were dozing over superficial anatomy in Britain, the French and German professors were diligently pursuing their elaborate researches. The spirit of John Hunter had departed from his own land, and animated Söemmering and Meckel, and Tiedemann, Beclard, the Cloquets, Breschet, and many whom we do not mention, because their names are in men's mouths. Anatomical science sank in this country in proportion as it rose abroad, and at last we became aware of the pass to which our pseudo-utilitarians were leading us.

We are, after all, an intellectual people, and the truth is seldom presented to us in vain.

We appreciate and seize it, and our national resolution and energy of purpose, guided by that common sense which is our peculiar characteristic, generally enable us to carry out our purpose to a successful issue. Dr. Knox's translation of M. Cloquet's System of Descriptive Anatomy appeared. It boldly proclaimed the necessity for extreme minuteness—the fallacy of saying that this fact was important and that was not—the danger of breeding such a laxity of mind in youth. The appeal was to the reason, and its triumph was equally signal and sudden. The teaching of anatomy was revolutionized in most of the schools—the cloud of manuals was dispersed—teachers who, on the old system, had been the most popular, were deserted—the teachers of the new became popular—and, to the astonishment of all who had been educated in the belief that minute anatomy was intolerable, it not only became bearable, but, by the students themselves, was most liked; such is the fact. Take the majority of medical students, and they *like* minute anatomy. There is something in it satisfactory to that spirit of curiosity which was implanted in our minds, for the purpose of enabling us to do what we have done in science and in art—in abstract science and the arts of life.

The work of Mr. Clark is an attempt to resuscitate the taste for the quid utile species of anatomy. That this spirit influences the author will be evident, from the motto which is to be found upon his title-page, as well as from one or two passages which we shall quote. The motto is taken from Cheselden.

"The study of anatomy," said that old and excellent surgeon, "as it leads to the knowledge of nature and the art of healing, needs not many tedious descriptions: what is most worth knowing is soonest learned, and least the subject of disputes; while dividing and describing the parts, more than the knowledge of their uses requires, perplexes the learner, and makes the science dry and difficult."

This sentiment of Cheselden is sufficiently expressive, and embodies the spirit of learning anatomy and teaching it, which may be said to be only just overthrown.

Mr. Clark assures us that his own motive in offering the present volume to the public was the following:—He felt the want of some assistance to draw his attention to practical points during his own early dissections of the nerves; and he is deeply impressed with the importance of combining the study of physiology with anatomy, a point much neglected in the dissecting-room: should he fail in his desire to supply, in some measure, this desideratum by the present manual, he will have to regret, in common with most unsuccessful authors, that his ability was not equal to his wishes.

A little farther on, his own opinions with respect to minute dissections are still more unequivocally stated. We extract a note to the brief introduction to the work.

"Previous to commencing the study of this branch of anatomy, let me warn the student against yielding too early to the desire of being *very accurate* in the more minute detail of various parts of the nervous system; I mean such detail as can have no evident points of either practical or physiological importance connected with it: for, let him be assured, such a course will most materially interfere with his acquiring a useful knowledge of his subject. Nor am I speaking at random; for every one who has been in the habit of attending much to the dissecting-room must have remarked the singular infatuation with which, in spite of all intreaty, some pupils will devote their whole time to unravelling and committing to memory a series of trifling points relative to the position of certain minute vessels and nerves, which it requires a great effort to retain, and which can, and will, never be turned to any useful account: and this all to the neglect of practical points of the first importance. Let the student, then, acquire first of all, what will stand him in good stead when he is practising his profession; and afterwards at his leisure he may become a more finished anatomist: for, however interesting much of the detail above alluded to may be, it is certainly not *essential* to the practitioner, and is very rarely retained except by those who are constantly dissecting. So true is the remark, that what is most useful is most easily acquired and remembered!"

It will easily be gathered from the tenor of our observations, and our readers, indeed, are very well aware, that we are at issue with Mr. Clark, and those who think with him on this subject. We are averse to enter again on a question, on which we have already and often touched; but we cannot refrain from briefly defending the views of the promoters of minute anatomy.

1. The study of anatomy must be considered as not merely the acquisition of a certain number of facts, to be subsequently applied to the solution of questions in the practice of medicine and surgery, but as a system of *education.* The habit of exact and minute investigation is excellent mental discipline. It qualifies for other investigations, and the young man who has been taught, and who has learnt, to observe closely where the advantage is not palpable, will be likely in after-life to carry the *habit* of application, of method, and of accuracy into his practical pursuits. The positive knowledge which it is possible for a student to acquire is, after all, inconsiderable. The clamour of the day would make him a sciolist. He is condemned in a brief period to run from subject to subject, and from lecture to lecture—he is told what is the smallest modicum he may be contented with in each department—and the grinder finishes the job by *veneering* the ill-jointed rickety upholstery.

2. It is easy to say that the student should confine himself to points of practical importance. But, when an excuse for indolence is offered, are students so zealous, is youth so calculating, that that will fail to be eagerly received? The neophyte will not stop at that nice point which the teacher so confidently talks of, and laxity of study, once encouraged, will be carried on by its own inertia. The young man is taught to ask himself incessantly—" Is it *necessary* that I should fatigue myself with learning this?" He is told to gauge his work by the uncertain rule of future need. Present convenience will find many and cogent reasons for clipping that work to the uttermost.

3. The student of to-day is the raw material of the teacher of to-morrow. If his own mode of education has been lax, what are we to expect from the one that he will inculcate? Remedies for moral, as for physical ills, are occasional and violent—the tendency to aggravation is gradual and sure. A bad system is the worst of evils, for nothing but revolution can remove it, and revolution, even when effectual, is always late. How long inaccurate anatomy was in request, and how continually it was becoming more inaccurate still, before the late sudden alteration took place! Should we again fall back to the vicious state in which we were, the *science* of anatomy in this country would be destroyed. Nothing but the most untiring exertions can repair what we have already lost, and restore us to equality with our neighbours.

4. When we are told to attend to practical points, we must first agree on what are practical. Supposing science limited, and our knowledge of it perfect, we might do so. But discovery is daily extending the sphere of the practical application of minute anatomical knowledge, and, for discovery, it is necessary that that knowledge be minute. Some years ago, the utilitarian might have said—" Why my young friends, harrass yourselves with making out the branches of the portio dura, and of the three divisions of the fifth nerve? There you see them expanded on the face—they go every where—supply it all—the broad fact is practically quite sufficient for you." But, when our young friends were consulted on a case of tic douloureux, or of paralysis of the portio dura, they must have felt, and they often did feel, much embarrassment. It is well known that Mr. John Bell proposed, in one case, to divide the divisions of the fifth where they emerge upon the face, in an instance of tic douloureux, concluding that the portio dura, being left, would be quite enough for practical purposes. Our increase of information has shewn us, that what was then thought practically sufficient is really not so; but it cannot tell us what is the precise limit of practical utility.

These remarks, hurried and imperfect as they are, are not directed against Mr. Clark, but are offered as a defence for those, who think that anatomy should be very minutely acquired, without reference to views of what may appear of immediate practical utility. Anatomy is the chief thing which the *student* has to learn. He has time to learn it well, and he ought to learn it well. He is sure afterwards to forget much of it, but he may be certain that, the more he acquires at first, the more he will retain at last.

When we turn from Mr. Clark's observations to the work itself, we cannot but applaud its execution. It is very superior to all the manuals of the old class, and to most of the present. We are personally unacquainted with the author, and can, therefore, have no motive, save the most correct one, for expressing our sense of the merits of his work. Whilst we differ from him in the general views that we have touched on, we feel great pleasure in publicly complimenting him on the modesty, industry, and discrimination, that he has evinced in the construction of this little volume. We beg to recommend it to our readers; we shall personally introduce it to our pupils.

